# The CaM Kinase CMK-1 Mediates a Negative Feedback Mechanism Coupling the *C*. *elegans* Glutamate Receptor GLR-1 with Its Own Transcription

**DOI:** 10.1371/journal.pgen.1006180

**Published:** 2016-07-27

**Authors:** Benjamin J. Moss, Lidia Park, Caroline L. Dahlberg, Peter Juo

**Affiliations:** 1 Department of Developmental, Molecular and Chemical Biology, Tufts University School of Medicine, Boston, Massachusetts, United States of America; 2 Graduate Program in Neuroscience, Sackler School of Graduate Biomedical Sciences, Tufts University School of Medicine, Boston, Massachusetts, United States of America; 3 Graduate Program in Cellular, Molecular and Developmental Biology, Sackler School of Graduate Biomedical Sciences, Tufts University School of Medicine, Boston, Massachusetts, United States of America; 4 Biology Department, Western Washington University, Bellingham, Washington, United States of America; Stanford University School of Medicine, UNITED STATES

## Abstract

Regulation of synaptic AMPA receptor levels is a major mechanism underlying homeostatic synaptic scaling. While *in vitro* studies have implicated several molecules in synaptic scaling, the *in vivo* mechanisms linking chronic changes in synaptic activity to alterations in AMPA receptor expression are not well understood. Here we use a genetic approach in *C*. *elegans* to dissect a negative feedback pathway coupling levels of the AMPA receptor GLR-1 with its own transcription. GLR-1 trafficking mutants with decreased synaptic receptors in the ventral nerve cord (VNC) exhibit compensatory increases in *glr-1* mRNA, which can be attributed to increased *glr-1* transcription. Glutamatergic transmission mutants lacking presynaptic *eat-4*/VGLUT or postsynaptic *glr-1*, exhibit compensatory increases in *glr-1* transcription, suggesting that loss of GLR-1 activity is sufficient to trigger the feedback pathway. Direct and specific inhibition of GLR-1-expressing neurons using a chemical genetic silencing approach also results in increased *glr-1* transcription. Conversely, expression of a constitutively active version of GLR-1 results in decreased *glr-1* transcription, suggesting that bidirectional changes in GLR-1 signaling results in reciprocal alterations in *glr-1* transcription. We identify the CMK-1/CaMK signaling axis as a mediator of the *glr-1* transcriptional feedback mechanism. Loss-of-function mutations in the upstream kinase *ckk-1*/CaMKK, the CaM kinase *cmk-1*/CaMK, or a downstream transcription factor *crh-1*/CREB, result in increased *glr-1* transcription, suggesting that the CMK-1 signaling pathway functions to repress *glr-1* transcription. Genetic double mutant analyses suggest that CMK-1 signaling is required for the *glr-1* transcriptional feedback pathway. Furthermore, alterations in GLR-1 signaling that trigger the feedback mechanism also regulate the nucleocytoplasmic distribution of CMK-1, and activated, nuclear-localized CMK-1 blocks the feedback pathway. We propose a model in which synaptic activity regulates the nuclear localization of CMK-1 to mediate a negative feedback mechanism coupling GLR-1 activity with its own transcription.

## Author Summary

Synaptic homeostasis increases or decreases synaptic strengths in order to stabilize neuronal firing in response to alterations in neuronal activity. Synaptic homeostasis plays an important role during neuronal development and may be deregulated in several neurological diseases. Neurons regulate glutamate neurotransmitter receptor levels at synapses to alter the strength of synaptic signaling during a form of homeostasis termed synaptic scaling. While many molecules have been implicated in synaptic scaling *in vitro* using cultured rodent neuron or slice preparations, the underlying *in vivo* mechanisms are not well understood. Here we use the genetic model organism *C*. *elegans* to identify *in vivo* mechanisms involved in a compensatory feedback pathway reminiscent of synaptic homeostasis that couples activity of the glutamate receptor GLR-1 with its own transcription. We show that *glr-1* transcription is regulated in a compensatory manner by bidirectional changes in synaptic activity. We find that the CMK-1/CaM kinase signaling pathway represses *glr-1* transcription. Furthermore, the subcellular distribution of CMK-1 between the cytoplasm and nucleus is regulated by GLR-1 and is important for mediating the feedback mechanism. This study uses genetics to dissect a negative feedback pathway *in vivo* and identifies the signaling mechanism that links changes in synaptic activity directly to *glr-1* transcription.

## Introduction

Homeostatic synaptic plasticity alters synaptic strengths in order to compensate for perturbations in neuronal activity. Homeostasis is thought to stabilize neuronal firing rates to remain within a physiological range in response to developmental changes in connectivity or alterations in synaptic strength during experience-dependent plasticity [1, 2]. Synaptic scaling is a form of homeostatic synaptic plasticity that has been widely studied *in vitro* [2–6] and *in vivo* after sensory deprivation in the rodent visual cortex [7–9].

One major mechanism underlying changes in synaptic strength during synaptic scaling is the regulation of AMPA receptor (AMPAR) levels at synapses. During homeostatic scaling, chronic activity-blockade or enhancement of activity results in compensatory increases or decreases, respectively, in AMPAR abundance at synapses. These changes in synaptic AMPARs are achieved, in part, by altering the rates of receptor exo- or endocytosis [3–6, 10–14].

Many molecules have been implicated in regulating synaptic AMPAR levels during homeostasis [11–13, 15–17]. In particular, homeostatic synaptic plasticity requires calcium signaling and the CaM kinases CaMKK and CaMKIV [3, 18–20]. Inhibition of calcium transients or CaMK signaling phenocopies activity-blockade and leads to increases in synaptic AMPARs [19]. Similarly, inhibition of voltage-gated calcium channels or CaMK signaling prevents scaling down of synaptic AMPARs [18]. Homeostatic synaptic plasticity is dependent on transcription, as pharmacological inhibition of transcription prevents bidirectional synaptic scaling [18, 19, 21, 22]. Interestingly, activity-blockade results in decreased levels of activated CaMKIV in the nucleus in a transcription-independent manner [19], suggesting that CaMKIV may translocate between the cytoplasm and nucleus during synaptic scaling to regulate transcription. These studies suggest that nuclear CaMKIV represses synaptic scaling and the associated increase in synaptic AMPARs in response to activity-blockade, but the transcriptional targets of CaMKIV responsible for the increase in synaptic AMPARs have not been defined.

Here we investigate a compensatory feedback pathway in *C*. *elegans* where synaptic levels of the AMPAR GLR-1 are negatively coupled to *glr-1* transcription via the CMK-1/CaMK signaling pathway. In *C*. *elegans*, CMK-1 is the sole ortholog of mammalian CaMKI and CaMKIV. As in mammals, CMK-1 is phosphorylated by CKK-1/CaMKK and can regulate CRH-1, the *C*. *elegans* homolog of CREB [23–25]. Recent studies in *C*. *elegans* show that CMK-1 can shuttle between the nucleus and cytoplasm to regulate temperature thresholds and experience-dependent thermotaxis under physiologic temperature and in response to noxious heat [26–28].

While much progress has been made identifying molecules involved in homeostatic synaptic scaling in neuronal and slice cultures [13], *in vivo* studies of mechanisms directly linking chronic changes in activity to regulation of AMPAR expression are lacking. Here we use a genetic approach to identify *in vivo* mechanisms involved in a negative feedback pathway in *C*. *elegans* that is reminiscent of synaptic homeostasis. We show that chronic activity-blockade or enhancement of GLR-1 function results in bidirectional changes in *glr-1* transcription *in vivo*. We find that regulation of *glr-1* transcription in response to chronic changes in synaptic activity requires the CMK-1 signaling pathway and redistribution of CMK-1 between the nucleus and cytoplasm. This study identifies the signaling mechanism underlying a compensatory feedback pathway that couples GLR-1 with its own transcription.

## Results

### *glr-1* transcription is negatively coupled to GLR-1 levels in the ventral nerve cord

We previously found that trafficking mutants with reduced GLR-1 abundance at synapses in the ventral nerve cord (VNC) exhibit reciprocal increases in *glr-1* mRNA levels. Specifically, animals with mutations in the deubiquitinating enzyme USP-46, which removes ubiquitin from GLR-1 and protects it from degradation, exhibit decreased levels of GLR-1 in the VNC and a compensatory 3 fold increase in *glr-1* transcript levels as measured by real-time quantitative PCR (RT-qPCR) [29]. Similarly, mutations in the kinesin motor KLP-4/KIF13, which positively regulates GLR-1 trafficking to the VNC, result in decreased levels of GLR-1 in the VNC and a compensatory 2–3 fold increase in *glr-1* transcript levels [30]. We hypothesized that GLR-1 levels or function at synapses in the VNC are monitored and coupled via a negative feedback mechanism to *glr-1* transcript levels.

To investigate the molecular mechanisms involved in this feedback pathway, we created a series of transgenic animals expressing different combinations of a nuclear-localized GFP reporter (NLS-tagged GFP fused to LacZ) under control of the *glr-1* promoter (*Pglr-1*) and/or the *glr-1* 3’ untranslated region (UTR). *Pglr-1* includes 5.3 kilobases of sequence upstream of the transcription start site [31] and allows monitoring of transcriptional activity of the promoter. The *glr-1* 3’UTR includes 100 base pairs downstream of the ORF, as predicted by modENCODE [32], and allows us to monitor the contribution of the 3’UTR to transcript levels.

**Fig 1:**
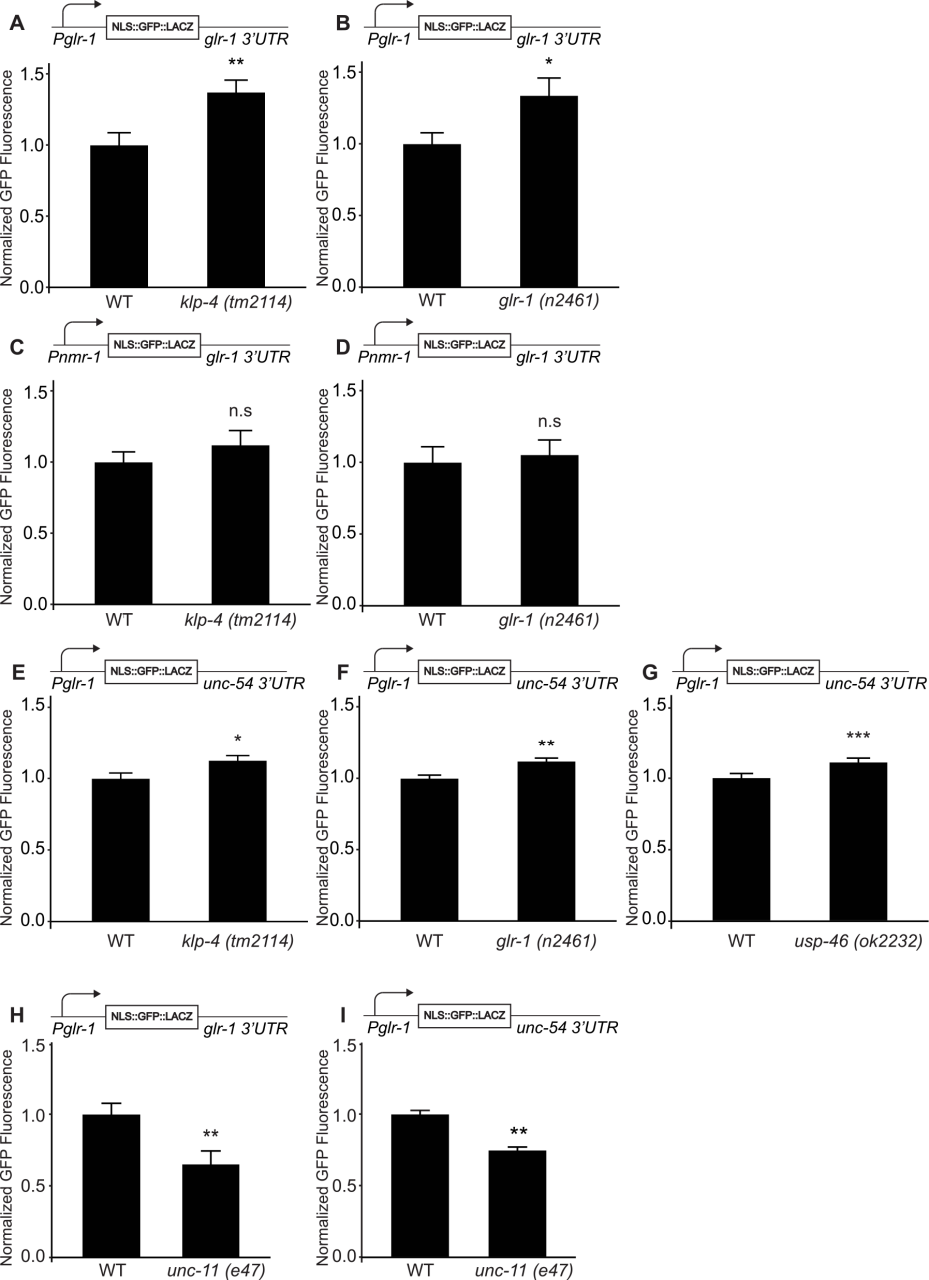
*glr-1* transcription is negatively coupled to GLR-1 levels in the VNC. (A-B) Mean GFP fluorescence (Normalized) of reporter *Pglr-1*::*NLS-GFP*::*LacZ*::*glr-1 3’UTR* in (A) wild type (n = 42) and *klp-4 (tm2114)* (n = 46) animals, and (B) wild type (n = 40) and *glr-1 (n2461)* (n = 42) animals is shown. (C-D) Mean GFP fluorescence (Normalized) of reporter *Pnmr-1*::*NLS-GFP*::*LacZ*::*glr-1 3’UTR* in (C) wild type (n = 76) and *klp-4 (tm2114)* (n = 58) animals, and (D) wild type (n = 35) and *glr-1 (n2461)* (n = 40) animals is shown. (E-G) Mean GFP fluorescence (Normalized) of reporter *Pglr-1*::*NLS-GFP*::*LacZ*::*unc-54 3’UTR* in (E) wild type (n = 20) and *klp-4 (tm2114)* (n = 20) animals, (F) wild type (n = 20) and *glr-1 (n2461)* (n = 20) animals, and (G) wild type (n = 31) and *usp-46 (ok2322)* (n = 31) animals is shown. (H) Mean GFP fluorescence (Normalized) of reporter *Pglr-1*::*NLS-GFP*::*LacZ*::*glr-1 3’UTR* in wild type (n = 39) and *unc-11 (e47)* (n = 24) animals is shown. (I) Mean GFP fluorescence (Normalized) of reporter *Pglr-1*::*NLS-GFP*:*LacZ*::*unc-54 3’UTR* in wild type (n = 41) and *unc-11 (e47)* (n = 41) animals is shown. For all reporter imaging, maximum GFP fluorescence was measured in the nucleus of the neuron PVC. Error bars represent SEM. Values that differ significantly from wild type are indicated by asterisks above each bar. The Student’s *t* test was used to compare means. * p < 0.05, ** p < 0.01, *** p < 0.001. n.s. denotes no significant difference (p > 0.05).

We first validated this *glr-1* reporter under control of both *Pglr-1* and the *glr-1* 3’UTR by testing if GFP fluorescence was altered in *klp-4*/KIF13 trafficking mutants. Briefly, we measured the maximum fluorescence intensity of GFP in the nucleus of the GLR-1-expressing interneuron PVC in wild type and *klp-4 (tm2114)* loss-of-function mutants (see Materials and Methods). We found that GFP fluorescence increased in *klp-4 (tm2114)* mutants (Fig 1A), consistent with our previous RT-qPCR results [30]. Because *klp-4* mutants have reduced GLR-1 at synapses in the VNC, this data implies that decreased synaptic GLR-1 may trigger a compensatory feedback pathway resulting in increased *glr-1* transcript. To directly test if loss of GLR-1 itself could trigger the feedback pathway, we measured the GFP reporter under control of *Pglr-1* and the *glr-1* 3’UTR in *glr-1* (*n2461*) null mutants. We found that GFP fluorescence increased in *glr-1* mutants to a similar extent as in *klp-4* mutants (Fig 1A and 1B). These data suggest that decreased GLR-1 protein or function is sufficient to trigger a compensatory feedback mechanism negatively coupling GLR-1 to its own transcript levels. These data also indicate that the *glr-1* promoter together with the *glr-1* 3’UTR are sufficient to mediate the feedback mechanism.

To determine the respective contributions of *Pglr-1* and the *glr-1* 3’UTR to the feedback mechanism, we generated additional GFP reporter transgenes consisting of NLS-GFP-LacZ under the control of either the *glr-1* or *nmr-1* promoters combined with either the *glr-1* or *unc-54* 3’UTRs. The *nmr-1* promoter provides an alternative promoter that is expressed in an overlapping set of neurons with GLR-1, including the interneuron PVC [33]. The *unc-54* 3’UTR is widely used for permissive gene expression in *C*. *elegans* [34]. We crossed these GFP reporter transgenes into several genetic backgrounds and measured GFP fluorescence in the nucleus of PVC interneurons as described above. When fluorescence was measured from a GFP reporter under control of the *nmr-1* promoter (*Pnmr-1*) and the *glr-1* 3’UTR, we observed no significant change in fluorescence in either *klp-4 (tm2114)* or *glr-1(n2461)* loss-of-function mutants (Fig 1C and 1D). This result suggests that the *glr-1* 3’UTR is not sufficient to mediate the feedback mechanism. On the other hand, when GFP fluorescence was measured from the reporter transgene containing *Pglr-1* and the *unc-54* 3’UTR (hereafter referred to as the *glr-1* transcriptional reporter), we observed a small but significant increase in fluorescence in both *klp-4* and *glr-1* mutants (Fig 1E and 1F). This *glr-1* transcriptional reporter was also increased in *usp-46 (ok2232)* loss-of-function mutants (Fig 1G), consistent with our previous RT-qPCR results [29]. Importantly, the *nmr-1* promoter and the *unc-54* 3’UTR are not regulated by the feedback pathway because a GFP reporter containing these elements was unaltered in *klp-4* and *glr-1* mutants (S1 Fig). Together, these data indicate that *Pglr-1* is sufficient to mediate the feedback mechanism, suggesting that neurons respond to decreased GLR-1 levels or function in the VNC by increasing *glr-1* transcription.

We next investigated whether the feedback mechanism was bidirectional by testing if increased GLR-1 in the VNC triggers a decrease in *glr-1* transcription. UNC-11/AP180 is a clathrin adaptin involved in endocytosis of GLR-1, and the receptor accumulates at the plasma membrane in the VNC of *unc-11* mutants [35]. We found that fluorescence of the GFP reporter under control of *Pglr-1* and the *glr-1* 3’UTR decreased in *unc-11(e47)* null mutants (Fig 1H). We observed a similar reduction of the *glr-1* transcriptional reporter in *unc-11* mutants (Fig 1I), suggesting that *Pglr-1* is sufficient to mediate decreased *glr-1* transcription. Interestingly, genetic double mutant analyses indicate that the effects of *unc-11* on *glr-1* transcription are not dependent on *glr-1* (S2 Fig). Together, these data suggest that mutation of the clathrin adaptin *unc-11*/AP180 likely blocks the endocytosis of another membrane protein or ion channel in addition to GLR-1, whose accumulation results in reduced *glr-1* transcription.

### Activity-dependent regulation of *glr-1* transcription

**Fig 2:**
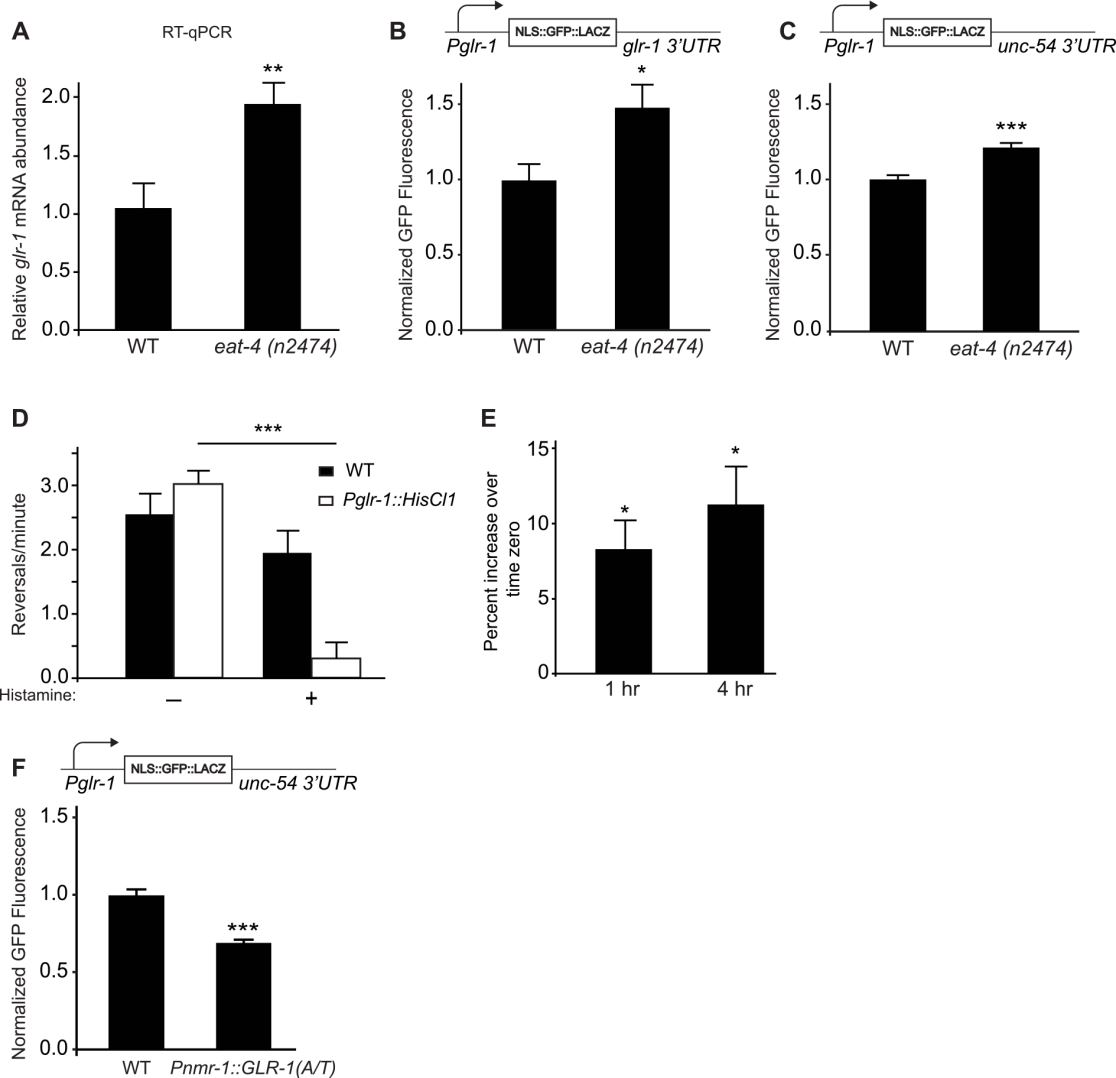
Activity-dependent regulation of *glr-1* transcription. (A) Real-time qPCR in wild type and *eat-4* (*n2474*) animals comparing *glr-1* expression in four biological replicates normalized to two references genes (*act-1* and *ama-1*). (B) Mean GFP fluorescence (Normalized) of reporter *Pglr-1*::*NLS-GFP*::*LacZ*::*glr-1 3’UTR* in wild type (n = 45) and *eat-4 (n2474)* (n = 40) animals is shown. (C) Mean GFP fluorescence (Normalized) of reporter *Pglr-1*::*NLS-GFP*::*LacZ*::*unc-54 3’UTR* in wild type (n = 37) and *eat-4 (n2474)* (n = 45) animals is shown. (D) Spontaneous reversals of wild type and HisCl1 (*Pglr-1*::*HisCl1*)-expressing animals on standard plates or those containing 10 mM histamine were recorded for five minutes. n = 8 for all conditions. (E) HisCl1-expressing animals were placed on plates with 10 mM histamine for one and four hours and mean GFP fluorescence of reporter *Pglr-1*::*NLS-GFP*::*LacZ*::*unc-54 3’UTR* was normalized to unexposed animals. n = 30 animals per condition. (F) Mean GFP fluorescence (Normalized) of reporter *Pglr-1*::*NLS-GFP*::*LacZ*::*unc-54 3’UTR* in wild type (n = 39) and animals expressing *Pnmr-1*::*GLR-1(A/T)* (n = 49) are shown. For all reporter imaging, maximum GFP fluorescence was measured in the nucleus of the neuron PVC. Error bars represent SEM. Values that differ significantly from wild type are indicated by asterisks above each bar, whereas other comparisons are marked by horizontal lines. Student’s *t* test was used to compare means. * p < 0.05, ** p < 0.01, *** p < 0.001.

We performed several experiments to test if changes in glutamate signaling, rather than levels of synaptic GLR-1, were sufficient to trigger the transcriptional feedback mechanism. First, we tested whether reductions in glutamatergic transmission could trigger the feedback mechanism by analyzing *glr-1* expression in *eat-4* synaptic transmission mutants. EAT-4 is a vesicular glutamate transporter (VGLUT) responsible for loading glutamate into synaptic vesicles [36, 37]. Loss of *eat-4* results in defects in glutamatergic transmission [37, 38] and a compensatory increase in synaptic GLR-1 in the VNC [10]. We found that *eat-4 (n2474)* loss-of-function mutants exhibit increased endogenous *glr-1* mRNA levels compared to wild type controls using RT-qPCR (Fig 2A). In support of this data, we found that *eat-4 (n2474)* mutants also exhibit increased GFP fluorescence from the reporter under control of *Pglr-1* and the *glr-1* 3’UTR (Fig 2B). Furthermore, *Pglr-1* was sufficient to mediate this effect because GFP fluorescence still increased in *eat-4 (n2474)* mutants expressing the *glr-1* transcriptional reporter (Fig 2C). Together, these data suggest that chronic decreases in glutamate signaling (Fig 2) or postsynaptic glutamate receptors (Fig 1) are sufficient to trigger the *glr-1* transcriptional feedback pathway.

We next investigated whether direct and more acute suppression of neuronal activity specifically in GLR-1-expressing neurons could trigger the feedback mechanism using a recently developed chemical genetic silencing strategy. Ectopic expression of a *Drosophila* histamine-gated chloride channel (HisCl1) in *C*. *elegans* neurons enables relatively acute repression of neuronal activity by exogenous histamine [39]. We generated transgenic animals expressing HisCl1 in GLR-1-expressing neurons (*Pglr-1*::*HisCl1*) and verified the efficacy of this silencing approach by measuring GLR-1-dependent locomotion reversal behavior. The frequency of spontaneous reversals is regulated by glutamatergic signaling, and mutants with reduced glutamatergic signaling (i.e., *glr-1* or *eat-4* mutants) exhibit decreased reversal frequencies [33, 35, 40]. We found that exposure of animals expressing HisCl1 to exogenous histamine for 10 minutes led to a dramatic decrease in spontaneous reversal frequency compared to wild type controls (Fig 2D). This data suggests that activation of HisCl1 channels specifically in GLR-1-expressing neurons suppresses their activity and impacts GLR-1-dependent locomotion behavior. In order to test whether direct inhibition of GLR-1-expressing neurons could increase *glr-1* transcription, we exposed HisCl1-expressing animals to histamine for one and four hours and then measured *Pglr-1* activity using the *glr-1* transcriptional reporter. Fluorescence at each time point was normalized to HisCl1-expressing animals in the absence of histamine (see Materials and Methods). We found a small increase in GFP reporter fluorescence after both one and four hours of histamine treatment (Fig 2E). Although the histamine-induced effect on the *glr-1* transcriptional reporter was modest, it was significant (p<0.05) and suggests that direct inhibition of GLR-1-expressing neurons can trigger an increase in *glr-1* transcription. In contrast, wild type animals not expressing HisCl1 showed no significant increase in *Pglr-1* activity when exposed to histamine (S3 Fig). We did, however, observe a reduction in *Pglr-1* activity in wild type animals after four hours of histamine exposure (S3 Fig). Unfortunately, this decrease in *Pglr-1* activity precluded our ability to test whether long term inhibition by histamine could also induce a late *glr-1* transcriptional response. Nevertheless, these results suggest that decreasing neuronal activity specifically in GLR-1-expressing neurons can trigger the feedback mechanism to increase *glr-1* transcription in the mature nervous system.

Finally, we investigated whether directly increasing GLR-1 function could regulate the transcriptional feedback pathway. We increased GLR-1 activity in a subset of interneurons by expressing a mutant version of GLR-1 (under control of the *nmr-1* promoter), that contains an alanine to threonine substitution (A/T) in the pore domain resulting in a constitutively active channel with increased conductance [40]. Animals expressing GLR-1(A/T) exhibit a dramatic increase in spontaneous locomotion reversals consistent with increased glutamatergic signaling [29, 40, 41]. We found that *Pglr-1* activity decreased in GLR-1(A/T)-expressing animals compared to wild type controls (Fig 2F). These data are consistent with the hypothesis that increased GLR-1 function triggers the feedback pathway to reduce *glr-1* transcription. Together, our data show that increasing or decreasing glutamatergic signaling results in compensatory and reciprocal changes in *glr-1* transcription.

### The CMK-1/CaMK signaling pathway regulates *glr-1* transcription

CaM kinases (CaMKs) I and IV are important mediators of calcium-dependent signaling mechanisms involved in neuronal development and function. In particular, CaMKIV can mediate activity-dependent regulation of gene transcription [42], and has been shown to mediate AMPAR-dependent homeostatic synaptic scaling in a transcription-dependent manner [18, 19]. In *C*. *elegans*, CMK-1, the homolog of CaMKI and CaMKIV [24], is widely expressed in the nervous system [26], and has been shown to function in specific sensory neurons to mediate experience-dependent thermotaxis at physiological temperatures and avoidance of noxious heat [26–28]. However, the downstream transcriptional targets of CMK-1 and CaMKIV that mediate their physiological effects are not clear.

**Fig 3:**
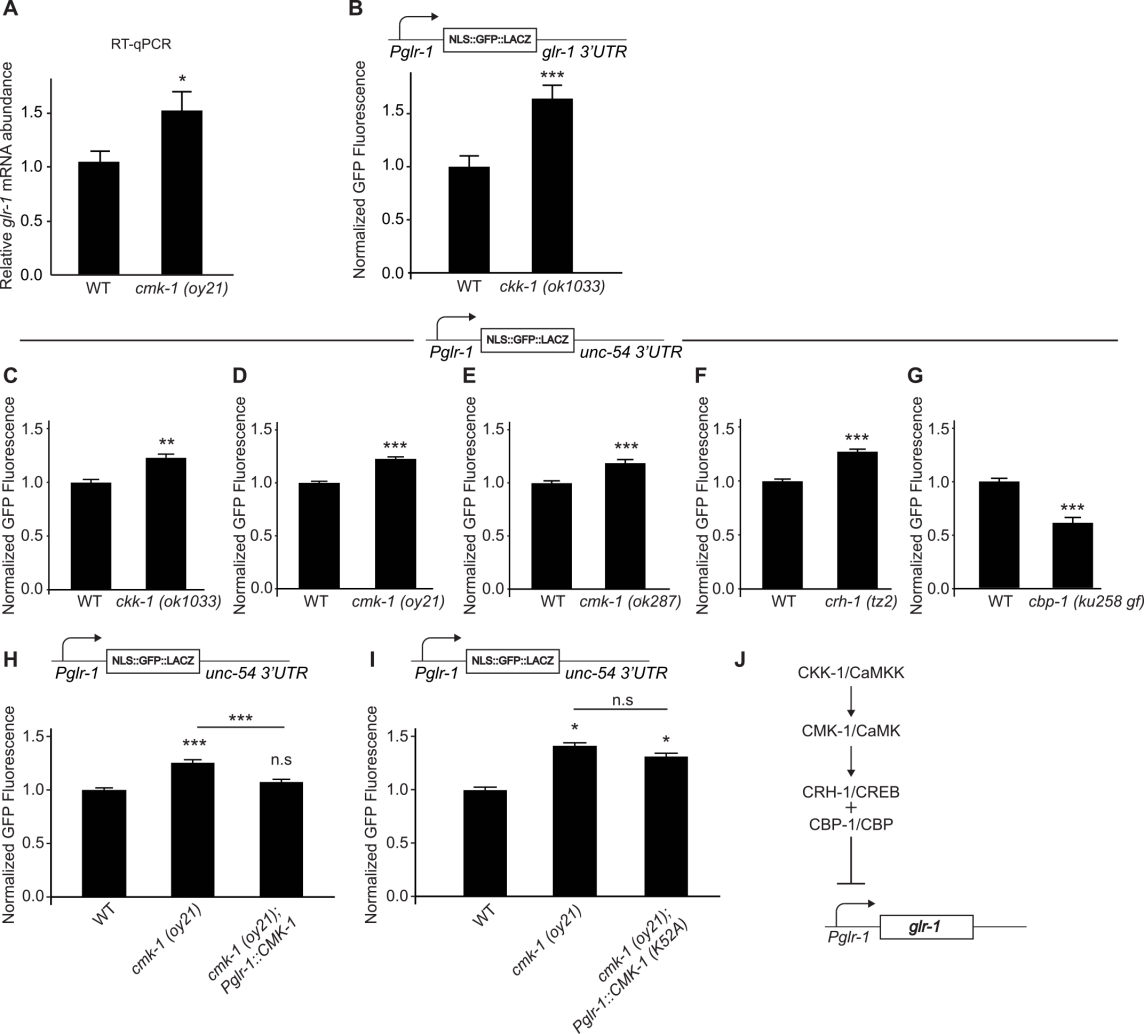
The CMK-1/CaMK signaling pathway regulates *glr-1* transcription. (A) Real-time qPCR in wild type and *cmk-1 (oy21)* animals comparing *glr-1* expression in four biological replicates normalized to two references genes (*act-1* and *ama-1*). (B) Mean GFP fluorescence (Normalized) of reporter *Pglr-1*::*NLS-GFP*::*LacZ*::*glr-1 3’UTR* in wild type (n = 45) and *ckk-1 (ok1033)* (n = 48) animals is shown. (C-G) Mean GFP fluorescence (Normalized) of reporter *Pglr-1*::*NLS-GFP*::*LacZ*::*unc-54 3’UTR* in wild type, (C) *ckk-1 (ok1033)*, (D) *cmk-1 (oy21)*, (E) *cmk-1 (ok287)*, (F) *crh-1 (tz2)*, and (G) *cbp-1 (ku258 gf)* animals is shown. n = 45 for all genotypes. (H) Mean GFP fluorescence (Normalized) of reporter *Pglr-1*::*NLS-GFP*::*LacZ*::*unc-54 3’UTR* was measured in wild type, *cmk-1 (oy21)* mutants, and *cmk-1 (oy21)* mutants animals expressing wild type CMK-1 (*Pglr-1*::*CMK-1)*. n = 64 for all genotypes. (I) Mean GFP fluorescence (Normalized) of reporter *Pglr-1*::*NLS-GFP*::*LACZ*::*unc-54 3’UTR* was measured in wild type (n = 41), *cmk-1 (oy21)* mutants (n = 28), and *cmk-1 (oy21)* mutants expressing a kinase-dead version of CMK-1 (*Pglr-1*::*CMK-1 (K52A))* (n = 44). (J) Model of the CMK-1/CaMK signaling pathway repressing *glr-1* transcription. For all reporter imaging, maximum GFP fluorescence was measured in the nucleus of the neuron PVC. Error bars represent SEM. Values that differ significantly from wild type are indicated by asterisks above each bar, whereas other comparisons are marked by horizontal lines. Student’s *t* test (A-G) or ANOVA with Tukey-Kramer *post hoc* test (H-I) were used to compare means. * p < 0.05, ** p < 0.01, *** p < 0.001. n.s. denotes no significant difference (p > 0.05).

To test whether CMK-1 was involved in regulating *glr-1* transcription, we first measured endogenous *glr-1* mRNA levels in *cmk-1 (oy21)* loss-of-function mutants using RT-qPCR. Intriguingly, we found increased *glr-1* mRNA levels relative to two reference genes (*act-1* and *ama-1*) in *cmk-1 (oy21)* mutants (Fig 3A), suggesting that CMK-1 negatively regulates *glr-1* transcript levels. Consistent with this result, loss-of-function mutations in *ckk-1*/CaMKK, the upstream activator of CMK-1, resulted in increased GFP fluorescence from a reporter under control of *Pglr-1* and the *glr-1* 3’UTR (Fig 3B). We next tested whether *Pglr-1* was sufficient to mediate the effects of the CMK-1 pathway using the *glr-1* transcriptional reporter. We found that *Pglr-1* activity increased in *ckk-1 (ok1033)* loss-of-function mutants (Fig 3C) and two independent loss-of-function alleles of *cmk-1* (*oy21* and *ok287*) (Fig 3D and 3E). These results indicate that the CMK-1 signaling pathway acts basally to repress *glr-1* transcription. Expression of *cmk-1* cDNA specifically in GLR-1-expressing neurons rescues the increase in *Pglr-1* activity observed in *cmk-1 (oy21)* loss-of-function mutants (Fig 3H), whereas expression of a kinase-dead version of CMK-1(K52A) [25] does not rescue (Fig 3I). The difference in rescue between the wild-type and kinase-dead versions of CMK-1 cannot be explained by different levels of transgene expression, as both *Pglr-1*::*CMK-1 and Pglr-1*::*CMK-1(K52A)* transgenes were expressed at comparable levels as determined by RT-qPCR (S4 Fig). These results indicate that CMK-1 functions in a kinase-dependent manner specifically in GLR-1-expressing neurons to repress *glr-1* transcription.

CaMKI and CaMKIV in mammals, and CMK-1 in *C*. *elegans*, have been shown to phosphorylate the transcription factor cyclic AMP response element binding protein (CREB) to regulate gene expression [24, 25, 43–46]. Thus, we tested whether mutations in *crh-1*, the *C*. *elegans* homolog of CREB, affected *glr-1* transcription. We found that fluorescence of the *glr-*1 transcriptional reporter was increased in *crh-1 (tz2)* loss-of-function mutants (Fig 3F), consistent with a role for CREB as a downstream target of CMK-1 in regulating *glr-1* transcription. Additionally, since CREB is known to function together with the transcriptional co-activator CREB binding protein (CBP-1)/p300 which can also be phosphorylated by CaMKIV [42, 47], we took advantage of a gain-of-function allele in *cbp-1 (ku258 gf)* [48] to test if *cbp-1* was involved in regulating *glr-1* transcription. We found that *cbp-1 (ku258 gf)* mutants exhibited decreased fluorescence of the *glr-*1 transcriptional reporter (Fig 3G). Together, these results indicate that the CaMK signaling axis, including CKK-1/CaMKK, CMK-1/CaMK, CRH-1/CREB and CBP-1/CBP act to repress *glr-1* transcription (Fig 3J).

### The CMK-1/CaMK signaling pathway mediates the *glr-1* transcriptional feedback mechanism

**Fig 4:**
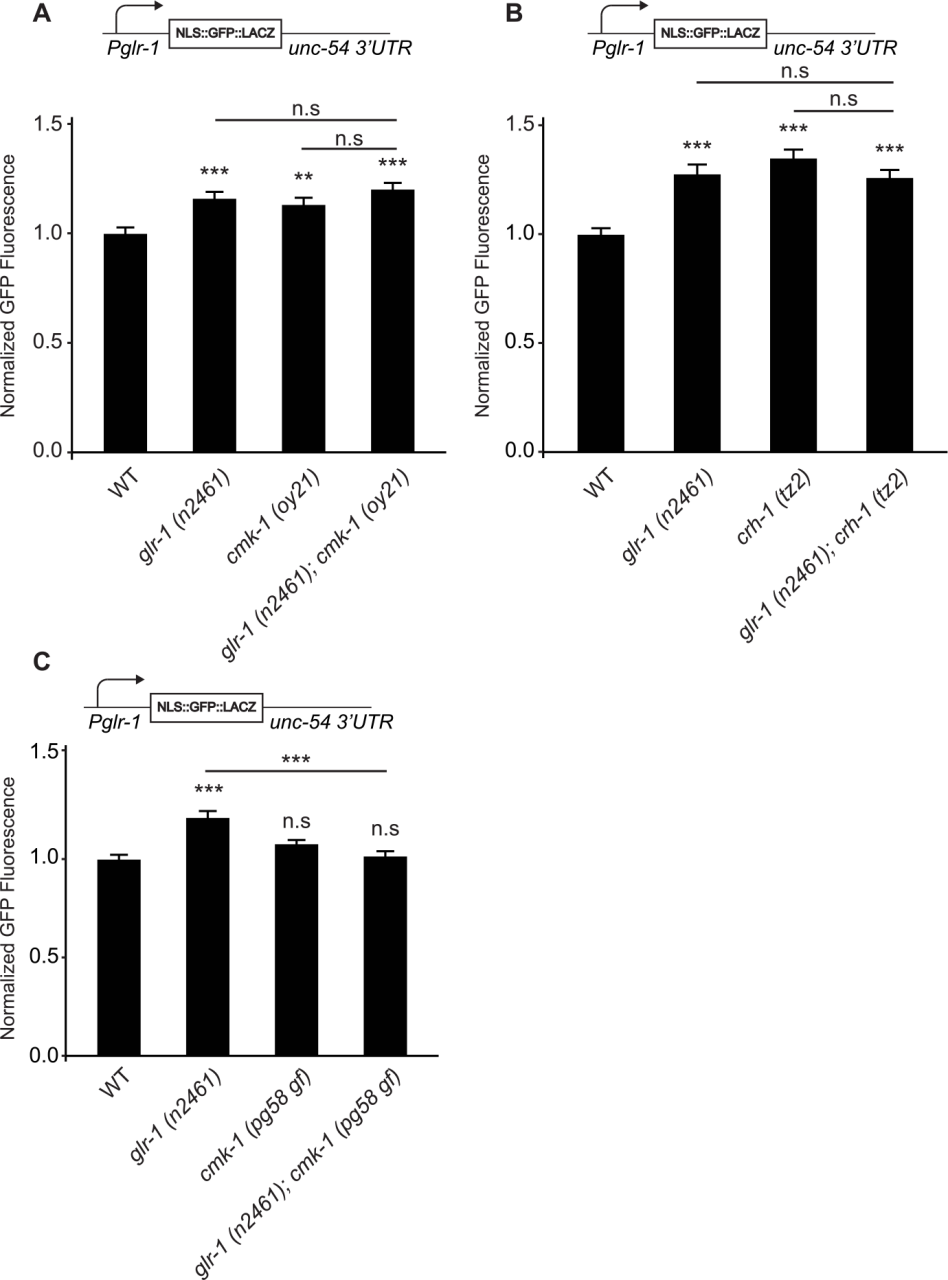
The CMK-1/CaMK signaling pathway mediates the *glr-1* transcriptional feedback mechanism. (A) Mean GFP fluorescence (Normalized) of reporter *Pglr-1*::*NLS-GFP*::*LacZ*::*unc-54 3’UTR* in wild type (n = 40), *glr-1 (n2461)* (n = 40), *cmk-1 (oy21)* (n = 41), and *glr-1 (n2461); cmk-1 (oy21)* (n = 35) animals is shown. (B) Mean GFP fluorescence (Normalized) of reporter *Pglr-1*::*NLS-GFP*::*LacZ*::*unc-54 3’UTR* in wild type, *glr-1 (n2461)*, *crh-1 (tz2)*, and *glr-1 (n2461); crh-1 (tz2)* animals is shown. n = 44 for all genotypes. (C) Mean GFP fluorescence (Normalized) of reporter *Pglr-1*::*NLS-GFP*::*LACZ*::*unc-54 3’UTR* in wild type, *glr-1 (n2461)*, *cmk-1 (pg58 gf)*, and *glr-1 (n2461); cmk-1 (pg58 gf)* animals is shown. n = 42 for all genotypes. Maximum GFP fluorescence was measured in the nucleus of the neuron PVC. Error bars represent SEM. Values that differ significantly from wild type are indicated by asterisks above each bar, whereas other comparisons are marked by horizontal lines. ANOVA with Tukey-Kramer *post hoc* test was used to compare means. **p<0.01, *** p < 0.001. n.s. denotes no significant difference (p > 0.05).

To test whether the negative feedback pathway triggered by loss of *glr-1* was mediated by CMK-1 signaling, we generated a series of genetic double mutants between *glr-1* and various CMK-1 pathway mutants. We hypothesized that if decreased GLR-1 signaling triggers an increase in *glr-1* transcription by deactivating the CMK-1 pathway, we would expect *glr-1*; *cmk-1* double mutants to have non-additive effects on the *glr-1* transcriptional reporter. Alternatively, if *cmk-1* functions in an independent pathway to regulate *glr-1* transcription, we would expect *glr-1*; *cmk-1* double mutants to have an additive effect on the *glr-1* transcriptional reporter. We found that *glr-1 (n2461)*; *cmk-1 (oy21)* double mutants exhibited an increase in the *glr-1* transcriptional reporter that is indistinguishable from either single mutant (Fig 4A). This non-additive effect is consistent with the idea that the *glr-1*-triggered feedback mechanism and *cmk-1* function in the same pathway to increase *glr-1* transcription. In support of this finding, we found that *glr-1 (n2461); crh-1 (tz2)* double mutants also exhibit an increase in the *glr-*1 transcriptional reporter that was identical to either single mutant (Fig 4B), suggesting that CRH-1/CREB also likely functions in the same pathway to negatively regulate *glr-1* transcription. Although these non-additive effects support the idea that the CMK-1 pathway may mediate the *glr-1* transcriptional feedback mechanism, we cannot formally rule out a potential ceiling effect of the reporter.

To provide further genetic evidence for a role for CMK-1 in the *glr-1* transcriptional feedback mechanism, we tested whether a recently isolated gain-of-function (*gf*) allele of *cmk-1*, *pg58*, could suppress the increase in *glr-1* transcription observed in *glr-1* mutants. *cmk-1 (pg58 gf)* contains a premature stop codon at W305 resulting in a truncated version of CMK-1 (1–304). CMK-1(1–304) is missing most of its regulatory domain and a putative nuclear export sequence (NES), and the altered protein has been shown to accumulate in the nucleus [27]. Interestingly, we found that although *cmk-1(pg58 gf)* did not affect basal *glr-*1 transcription, this gain-of-function allele completely blocked the increase in the *glr-*1 transcriptional reporter triggered by loss of *glr-1* (Fig 4C). Together, these data are consistent with the model that CMK-1 signaling mediates the *glr-1* transcriptional feedback mechanism.

### GLR-1 signaling regulates the nuclear localization of CMK-1

**Fig 5:**
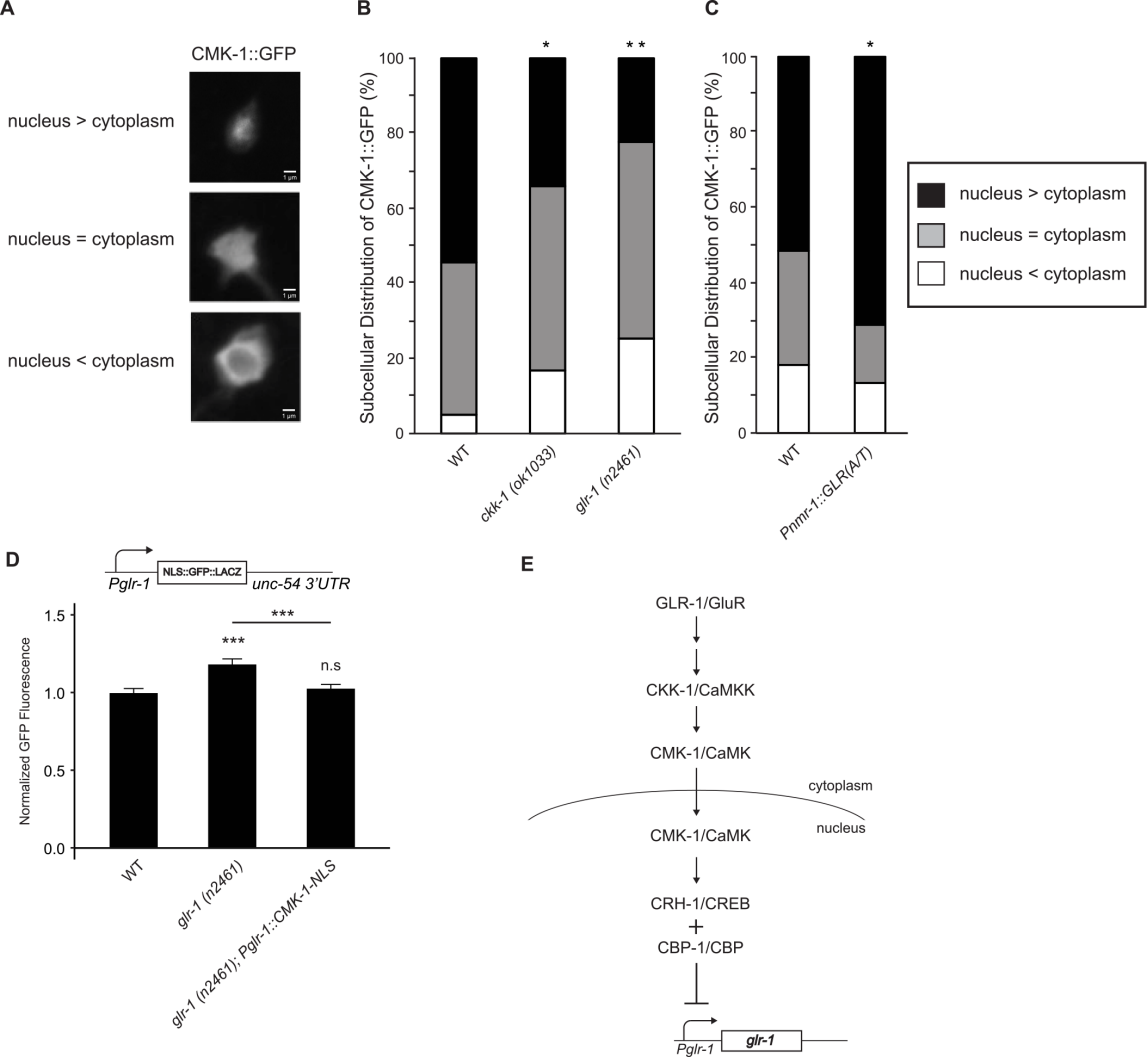
The nucleocytoplasmic distribution of CMK-1 is altered by GLR-1 signaling. (A) Representative confocal images of CMK-1::GFP in PVC neuronal cell bodies are shown illustrating three patterns of nucleocytoplasmic distribution of CMK-1::GFP: nucleus > cytoplasm, nucleus = cytoplasm and nucleus < cytoplasm. (B) The nucleocytoplasmic distribution of CMK-1::GFP in wild type (n = 64), *ckk-1 (ok1033)* (n = 47) and *glr-1 (n2461)* (n = 40) animals was scored by an experimenter blind to the genotypes and graphed. Comparison to wild type was made using the Chi-squared *post hoc* test. (C) The nucleocytoplasmic distribution of CMK-1::GFP in wild type (n = 112) and animals expressing *Pnmr-1*::*GLR-1(A/T)* (n = 59) were scored by an experimenter blind to the genotypes and graphed. Comparison to wild type was made using the Chi-squared *post hoc* test. (D) Mean GFP fluorescence (Normalized) of reporter *Pglr1*::*NLS-GFP*::*LacZ*::*unc-54 3’UTR* was measured in wild type (n = 19), *glr-1 (2461)* (n = 21), and *glr-1* (n2461) mutants expressing a nuclear-localized version of CMK-1 (*Pglr-1*::*CMK-1*::*EGL-13-NLS*) (n = 21). (E) Model showing the CMK-1/CaMK signaling pathway repressing *glr-1* transcription in the nucleus. Increased GLR-1 signaling leads to activation of CKK-1/CaMKK, which phosphorylates CMK-1/CaMK resulting in its translocation into the nucleus. Nuclear CMK-1 can then activate CRH-1/CREB and CBP-1/CBP to repress *glr-1* transcription. The lack of canonical CREB binding sites in the *Pglr-1* suggests that CREB likely indirectly regulates *glr-1* transcription. Maximum GFP fluorescence in (D) was measured in the neuron PVC. Error bars represent SEM. Values that differ significantly from wild type are indicated by asterisks above each bar, whereas other comparisons are marked by an horizontal line. ANOVA with Tukey-Kramer *post hoc* test was used to compare means. *** p < 0.001. n.s. denotes no significant difference (p > 0.05).

CaM kinases are well-known mediators of activity-dependent gene expression, and specific isoforms have been shown to translocate between the cytoplasm and nucleus [42, 49]. For example, in mammalian neuronal cultures, homeostatic increases in synaptic GluRs are correlated with a reduction in activated CaMKIV in the nucleus [19]. In *C*. *elegans*, CMK-1 can shuttle between the cytoplasm and nucleus to regulate thermosensory behaviors [27, 28]. Thus, we tested whether alterations in *glr-1* transcription were accompanied by changes in the subcellular localization of CMK-1. We expressed GFP-tagged CMK-1 (CMK-1::GFP) [26] in GLR-1-expressing interneurons and used confocal microscopy to determine the relative subcellular localization of CMK-1::GFP in the cytoplasm versus nucleus of PVC neurons (see Materials and Methods). The subcellular localization of CMK-1::GFP is regulated by changes in physiological temperature and noxious heat [27, 28], and CMK-1::GFP can rescue heat avoidance behavioral defects in *cmk-1* mutants, suggesting that the tagged protein is functional [27]. Since CKK-1 phosphorylation of CMK-1 has been shown to promote the nuclear accumulation of CMK-1::GFP in sensory neurons [27, 28], we first analyzed the subcellular localization of CMK-1::GFP (Fig 5A) in GLR-1-expressing neurons in *ckk-1* (*ok1033*) loss-of-function mutants. We found that CMK-1::GFP decreases in the nucleus and increases in the cytoplasm in *ckk-1* (*ok1033*) mutants (Fig 5B). In other words, the subcellular localization of CMK-1::GFP shifts from the nucleus towards the cytoplasm in *ckk-1* mutants, consistent with previous studies [27, 28].

To test whether the subcellular localization of CMK-1 is regulated by GLR-1 signaling, we analyzed the distribution of CMK-1::GFP in *glr-1* mutants. Similar to *ckk-1* mutants, we found that CMK-1::GFP decreases in the nucleus and increases in the cytoplasm in *glr-1* (*n2461*) mutants (Fig 5B). These results are consistent with the idea that decreased synaptic GLR-1 results in increased retention of CMK-1 in the cytoplasm and relief of repression of *glr-1* transcription. In contrast, we found that increasing GLR-1 signaling by expression of constitutively active GLR-1(A/T) in interneurons results in increased localization of CMK-1::GFP to the nucleus (Fig 5C). Together, these data suggest that increased or decreased GLR-1 signaling in interneurons results in increased or decreased accumulation, respectively, of CMK-1 in the nucleus.

To specifically test whether nuclear localization of CMK-1 is sufficient to repress the increase in *glr-1* transcription triggered by loss of glutamatergic signaling, we expressed a constitutively nuclear-localized version of CMK-1 containing an exogenous NLS (*Pglr-1*::*CMK-1*::*EGL-13-NLS*) in GLR-1-expressing neurons. CMK-1::EGL-13-NLS was shown to be five-fold enriched in the nucleus where it can rescue *cmk-1* null mutants for several thermosensory defects [28]. We found that expression of constitutively nuclear CMK-1 was sufficient to block the increase in the *glr-1* transcriptional reporter observed in *glr-1* (*n2461*) mutants (Fig 5D). These data suggest that nuclear localization of CMK-1 represses *glr-1* transcription and provides further evidence that the CMK-1 signaling pathway mediates the *glr-1* transcriptional feedback mechanism (Fig 5E).

## Discussion

Regulation of synaptic AMPAR levels mediates the homeostatic response to chronic changes in neuronal activity during synaptic scaling. The underlying mechanisms involved have largely been attributed to changes in AMPAR trafficking based on a variety of *in vitro* neuronal models [2, 13]. However, synaptic scaling can also regulate AMPAR expression and although synaptic scaling can be blocked by pharmacological inhibitors of transcription [18, 19, 21, 22], little is known about the *in vivo* mechanisms that link chronic changes in activity with regulation of AMPA receptor transcription. This study investigates a compensatory feedback mechanism in *C*. *elegans* reminiscent of synaptic homeostasis where synaptic GLR-1 is negatively coupled to its own transcription

### A negative feedback pathway couples GLR-1 with its own transcription

We found that GLR-1 trafficking mutants (i.e., *klp-4*/KIF13 or *usp-46* mutants) with decreased GLR-1 in the VNC exhibit compensatory increases in *glr-1* expression (Fig 1). Analysis of fluorescent reporters containing either *Pglr-1* or the *glr-1* 3’UTR revealed that the *glr-1* promoter was sufficient to mediate the feedback mechanism (Fig 1). Interestingly, although the *glr-1* 3’UTR alone did not appear to be sufficient to mediate the feedback pathway (Fig 1C and 1D), we noticed that reporter constructs containing the *glr-1* 3’UTR together with *Pglr-1* (Figs 1A, 1B, 1H and 3B) appear to have larger magnitude effects versus the *unc-54* 3’UTR (Figs 1E, 1F, 1I and 3C) hinting at a potential contribution of the *glr-1* 3’UTR. Statistical comparison of the relevant data sets revealed significant contributions (p<0.05, Two-way ANOVA) of the *glr-1* 3’UTR (together with *Pglr-1*) in *klp-4* (p = 0.03) and *ckk-1* (p = 0.03) mutant backgrounds. The contribution of the *glr-1* 3’UTR versus the *unc-54* 3’UTR in *glr-1* (p = 0.1) and *unc-11* (p = 0.2) mutant backgrounds did not reach statistical significance. Thus, the *glr-1* 3’UTR appears to contribute to the regulation of *glr-1* expression in the feedback pathway in some genetic backgrounds. A more detailed analysis of the *glr-1* 3’UTR together with other endogenous regulatory elements is warranted to fully understand the role of the *glr-1* 3’UTR in the feedback pathway. Interestingly, a recent study in rodent hippocampal neurons showed that microRNA miR-92A inhibits translation of GluA1 by binding to its 3’UTR, and that this miRNA-mediated mechanism regulates homeostatic scaling in response to chronic activity-blockade [50]. However, we did not find any conserved miRNA binding sites in the *glr-1* 3’UTR using several target site prediction algorithms. Furthermore, we found that the *glr-1* 3’UTR alone was not sufficient to mediate the feedback mechanism in *C*. *elegans* (Fig 1C and 1D). Thus, while non-conserved miRNAs may still contribute to the regulation of the *glr-1* 3’UTR, this regulation does not appear to be sufficient to mediate the feedback pathway.

We also investigated whether changes in glutamate signaling could trigger the feedback mechanism. We found that glutamatergic transmission mutants lacking *glr-1* itself (Fig 1) or the presynaptic *eat-4*/VGLUT (Fig 2) were sufficient to trigger the *glr-1* transcriptional feedback mechanism. Furthermore, expression of a constitutively active GLR-1, GLR-1(A/T), resulted in decreased *glr-1* transcription (Fig 2F). These data indicate that bidirectional changes in GLR-1 signaling are negatively coupled to *glr-1* transcription.

A previous study showed that chronic activity-blockade in *eat-4*/VGLUT mutants results in a homeostatic compensatory increase in synaptic GLR-1 levels that is mediated by changes in clathrin-mediated endocytosis [10]. We found that *eat-4* mutants also exhibit increased endogenous *glr-1* transcript based on RT-qPCR and increased *Pglr-1* activity based on a *glr-1* transcriptional reporter expressing nuclear-localized NLS-GFP-LacZ (Fig 2). Given the multiple mechanisms that contribute to synaptic scaling in mammalian neurons, we suspect that the homeostatic compensatory increase in GLR-1 observed in *eat-4* mutants is likely mediated by several mechanisms including changes in both transcription and trafficking of GLR-1.

### CMK-1/CaMK signaling mediates the feedback pathway

*In vitro* studies using rodent neuron or slice cultures showed that the CaMKIV signaling pathway regulates bidirectional synaptic scaling [18, 19]. In *C*. *elegans*, *cmk-1* is the only homolog of mammalian CaMKI and CaMKIV and shares features with both kinases. While the primary sequence of CMK-1 shows more homology to mammalian CaMKI, CMK-1 appears to function more like CaMKIV based on its neuronal expression pattern, its ability to phosphorylate CREB, and its localization to both the cytoplasm and nucleus [23–25, 51]. Our data show *in vivo* that the CMK-1/CaMK signaling pathway mediates the feedback mechanism and acts in the nucleus to repress *glr-1* transcription (Figs 3–5). We showed that *cmk-1* loss-of-function mutants had increased *glr-1* transcript levels based on RT-qPCR and fluorescent reporters (Fig 3). Analysis of a *glr-1* transcriptional reporter in CMK-1 signaling pathway mutants including *ckk-*/CaMKK*1*, *cmk-1*/CaMK, *crh-1*/CREB and *cbp-1*/CBP indicates that the CMK-1 signaling pathway represses *glr-1* transcription (Fig 3). Furthermore, rescue experiments indicate that CMK-1 functions in GLR-1-expressing neurons to repress *glr-1* transcription, and this effect is dependent on its kinase activity (Fig 3H and 3I).

Several pieces of evidence suggest that in addition to repressing basal *glr-1* transcription, CMK-1 also mediates the *glr-1* transcriptional feedback mechanism. First, analysis of genetic double mutants between *cmk-1* signaling pathway components and *glr-1* showed non-additive effects on *glr-1* transcription (Fig 4), consistent with the idea that CMK-1 signaling functions in the same pathway as the feedback mechanism triggered by loss of *glr-1*. Second, the feedback mechanism triggered by loss of *glr-1* or by expression of constitutively active GLR-1(A/T) regulated the subcellular distribution of CMK-1 between the cytoplasm and nucleus (Fig 5). These bidirectional changes in GLR-1 signaling had opposite effects on CMK-1 localization to the nucleus, consistent with the idea that decreased GLR-1 signaling results in decreased translocation of CMK-1 to the nucleus whereas increased GLR-1 signaling results in increased translocation of CMK-1 into the nucleus. Third, a gain-of-function allele (*pg58*) of *cmk-1* missing its NES and autoinhibitory domain [27] blocked the *glr-1* transcriptional feedback mechanism (Fig 4C). Furthermore, addition of an exogenous NLS to CMK-1, which forces CMK-1 into the nucleus [28], was sufficient to inhibit the *glr-1* transcriptional feedback pathway (Fig 5D). Together, these data are consistent with a model whereby increased synaptic GLR-1 activates the CMK-1 signaling pathway resulting in increased nuclear accumulation of CMK-1 and repression of *glr-1* transcription (see model in Fig 5E).

A recent study by Ma et al., (2014) using cultured rodent neurons showed that activation of nuclear CaMKIV and phosphorylation of CREB in response to acute stimulation is mediated by the nuclear translocation of γCaMKII [49]. Interestingly, γCaMKII functions in a kinase-independent manner as a shuttle to transport CaM into the nucleus to activate CaMKK and CaMKIV. In contrast, and consistent with previous studies in *C*. *elegans* reporting nuclear translocation of CMK-1 in sensory neurons [27, 28], our results show that CMK-1 translocates into the nucleus (Fig 5) and regulates *glr-1* transcription in a kinase-dependent manner (Fig 3). Although Ma et al. (2014) did not investigate the role of γCaMKII in activating CaMKIV in response to chronic changes in activity, our study suggests that mechanisms of activation of nuclear CaMK may differ between mammals and *C*. *elegans*. It will be interesting to test whether chronic changes in activity during synaptic scaling in mammalian neurons also require nucleocytoplasmic shuttling of CaM by γCaMKII.

Our results suggest that CMK-1 regulates *glr-1* transcription both basally and in response to changes in activity. We found that *glr-1* transcription increases in *cmk-1* signaling pathway mutants (Fig 3), suggesting that a low level of CMK-1 activity is required to basally repress *glr-1* transcription. However, manipulations that increased CMK-1 activity (i.e., *cmk-1(pg58 gf)* mutants) were not sufficient to repress basal *glr-1* transcription, but interestingly, could completely block the increased *glr-1* transcription triggered by loss of *glr-1* (Fig 4C). This effect of *cmk-1(pg58 gf)* is reminiscent of a previous finding in which the gain-of-function allele had no effect on basal secretion of neuropeptides from FLP thermosensory neurons but completely blocked heat-induced secretion of neuropeptides [27]. Together, these studies suggest that CMK-1 regulation of basal responses versus activity-induced responses may be differentially controlled.

### Transcriptional regulation of GluRs

With the exception of a recent report which showed that nuclear Arc represses GluA1 transcription during synaptic scaling [52], little is known about direct regulation of AMPAR transcription by chronic changes in activity. While several studies have shown that AMPAR mRNA and protein levels are altered during scaling [15, 53, 54] and synaptic scaling depends on CaMKIV and transcription [18, 19, 21, 22, 55], a direct connection between the CaMK pathway and AMPAR transcription has not been shown. In this study, we showed that bidirectional changes in synaptic activity regulate *glr-1* promoter activity in a reciprocal manner and that this effect is mediated by the CaMKK-CaMK signaling pathway.

We found that in addition to mutations in *ckk-1*/CaMKK and *cmk-1*/CaMK, mutations in *crh-1*/CREB or *cbp-1*/CBP also affect *glr-1* transcription (Fig 3). Since mammalian CaMKIV and *C*. *elegans* CMK-1 can phosphorylate and activate CREB and CRH-1, respectively [24, 25, 43–46], these data suggest that the CaMK-CREB axis represses *glr-1* transcription. However, this regulation is likely to be indirect because the *glr-1* promoter does not contain any canonical CREB binding sites, suggesting that CMK-1 may first activate CRH-1/CREB which in turn regulates transcription of a repressor that controls *glr-1* transcription.

Interestingly, two recent studies implicate global changes in DNA methylation as a mechanism to regulate AMPAR expression during synaptic scaling [56, 57]. These studies show that in cultured rodent neurons there is an overall reduction in DNA methylation in response to activity-blockade, whereas DNA methylation increases in response to enhanced neuronal activity. As methylation is typically associated with gene repression, these papers suggest that gene expression increases during synaptic scaling in response to activity-blockade and vice versa. Although the existence of DNA cytosine methylation is controversial in *C*. *elegans*, a recent paper showed that adenine methylation and the relevant modifying enzymes do exist in *C*. *elegans* and function to regulate transgenerational epigenetic changes [58]. It will be interesting in the future to test if DNA methylation is regulated by CMK-1 signaling to control the *glr-1* transcriptional feedback pathway.

In conclusion, we identified a novel compensatory feedback mechanism in *C*. *elegans* that couples GLR-1 glutamate receptors with their own transcription. We characterized this pathway *in vivo* and showed that CMK-1 represses *glr-1* transcription and translocates between the cytoplasm and nucleus to mediate the feedback mechanism. Regulation of *glr-1* transcription in *C*. *elegans* and GluR transcription in mammals in response to chronic changes in activity are poorly understood. Future studies are warranted to identify the relevant transcription factors that regulate *glr-1* transcription both basally and in response to changes in synaptic activity.

## Materials and Methods

### Strains

All strains were maintained at 20°C as previously described [59]. The following strains were used for this study:

N2

FJ1211 *pzEx329* [*Pglr-1*::*NLS-GFP*::*LacZ*::*glr-1 3’UTR*]

FJ1217 *pzEx329; glr-1 (n2461)*

FJ1374 *pzEx329; klp-4 (tm2114)*

FJ1268 *pzEx354* [*Pnmr-1*::*NLS-GFP*::*LacZ*::*glr-1 3’UTR*]

FJ1284 *pzEx354; glr-1 (n2461)*

FJ1271 *pzEx354; klp-4 (tm2114)*

FJ1047 *pzIs29* [*Pglr-1*::*NLS-GFP*::*LacZ*::*unc-54 3’UTR*]

FJ1109 *pzIs29; glr-1 (n2461)*

FJ1073 *pzIs29; klp-4 (tm2114)*

FJ1375 *pzIs29; usp-46 (ok2322)*

FJ1148 *pzIs29; unc-11 (e47)*

MT6318 *eat-4 (n2474)*

FJ1322 *pzEx329; eat-4 (n2474)*

FJ1237 *pzIs29; eat-4 (n2474)*

FJ1316 *pzEx362* [*Pglr-1*::*HisCl1*]

FJ1352 *pzIs29; pzEx362* [*Pglr-1*::*HisCl1*]

PY1589 *cmk-1 (oy21)*

VC691 *ckk-1(ok1033)*

FJ1291 *pzEx329; ckk-1 (ok1033)*

FJ1159 *pzIs29; ckk-1 (ok1033)*

FJ1141 *pzIs29; cmk-1 (oy21)*

VC220 *cmk-1 (ok287)*

FJ1376 *pzIs29; cmk-1 (ok287)*

YT17 *crh-1 (tz2)*

FJ1167 *pzIs29; crh-1 (tz2)*

MH2430 *cbp-1 (ku258)*

FJ1288 *pzIs29; cbp-1 (ku258)*

FJ1244 *pzIs29; cmk-1 (oy21); pzEx333* [*Pglr-1*::*CMK-1*]

FJ1222 *pzIs29; pzEx333* [*Pglr-1*::*CMK-1*]

FJ1235 *pzIs29; cmk-1 (oy21); pzEx338* [*Pglr-1*::*CMK-1 (K52A)*]

FJ1142 *pzIs29; glr-1 (n2461); cmk-1 (oy21)*

FJ1214 *pzIs29; glr-1 (n2461); crh-1 (tz2)*

GN244 *cmk-1 (pg58)* (Gift from Miriam Goodman and Dominique Glauser)

FJ1355 *pzIs29; cmk-1 (pg58)*

FJ1356 *pzIs29; glr-1 (n2461); cmk-1 (pg58)*

FJ1310 *pzIs29; unc-11 (e47); ckk-1 (ok1033)*

FJ1272 *pzEx356* [*Pglr-1*::*CMK-1*::*GFP*]

FJ1274 *pzEx356; ckk-1 (ok1033)*

FJ1273 *pzEx356; glr-1 (n2461)*

FJ1364 *pzEx356; unc-11 (e47)*

FJ1354 *pzIs29; glr-1 (n2461); pzEx370* [*Pglr-1*::*CMK-1*::*EGL-13 NLS*]

FJ1246 *pzEx342* [*Pnmr-1*::*NLS-GFP*::*LacZ*::*unc-54 3’UTR*]

FJ1377 *pzEx342; klp-4 (tm2114)*

FJ1247 *pzEx342; glr-1 (n2461)*

FJ1347 *pzEx329; unc-11 (e47)*

VM3898 *akEx52* [*Pnmr-1*::*GLR-1(A/T);lin-15(+)*];*lin-15(n765ts)* (Gift from Villu Maricq)

### Constructs

Plasmids were generated using standard recombinant DNA techniques, and transgenic strains were created by plasmid microinjection.

*Pglr-1*::*NLS-GFP*::*LACZ*::*unc-54 3’UTR* was made by cloning 5.3 kb upstream of the *glr-1* transcription start site into pPD96.04 (Addgene, Fire Lab *C*. *elegans* Vector Kit) containing *NLS-GFP*::*LACZ* to generate plasmid FJ#119 and injected at 50 ng/ul to make *pzEx260*, which was integrated to make *pzIs29*.

Pglr-1::NLS-GFP::LACZ::*glr-1 3’UTR* was made by PCR of the *glr-1 3’UTR* from CR3 and cloning into pV6 with Sac1 and Spe1 to make pBM7 and then PCR of *NLS-GFP*::*LACZ* from *Pglr-1*::*NLS-GFP*::*LACZ*::*unc-54 3’UTR* and cloning into pBM7 with Nhe1. pBM12 was injected at 60 ng/ul to make *pzEx329*.

*Pnmr-1*::*NLS-GFP*::*LACZ*::*glr-1 3’UTR* was made by digesting Pnmr-1 from pKM05 and swapping into pBM12 for *Pglr-1* with Sph1 and BamH1. pBM17 was injected at 50 ng/ul to make *pzEx354*.

*Pnmr-1*::*NLS-GFP*::*LACZ*::*unc-54 3’UTR* was made by digesting *Pnmr-1* from pKM05 and swapping into *Pglr-1*::*NLS-GFP*::*LACZ*::*unc-54 3’UTR* for *Pglr-1* with Sph1 and BamH1. pBM16 was injected at 50 ng/ul with *Pmyo-2*::*mCherry* at 10 ng/ul to make *pzEx342*.

*Pglr-1*::*HisCl1* was made by digesting pNP403 (*Ptag-168*::*HisCl1*::*SL2*::*GFP*) (Gift from Cori Bargmann) with Nhe1 and Kpn1 and cloning into pV6. pBM29 was injected at 5 ng/ul with Pmyo2::mCherry at 10 ng/ul to make *pzEx358*.

*Pglr-1*::*CMK-1*::*GFP* was made by PCR of *CMK-1*::*GFP* from *Pttx-1*::*CMK-1*::*GFP* (Gift from Piali Sengupta) and cloning into pV6 with Kpn1 and Sac1. pBM15 was injected at 2.5 ng/ul with Pmyo2::mCherry at 10 ng/ul to make *pzEx356*.

*Pglr-1*::*CMK-1* was made by PCR of CMK- from *Pttx-1*::*CMK-1*::*GFP* (Gift from Piali Sengupta) adding a 3’ stop codon and cloning into pV6 with Nhe1 and Kpn1. pBM13 was injected at 25 ng/ul with *Pmyo2*::*mCherry* at 10 ng/ul to make *pzEx334*.

*Pglr-1*::*CMK-1 (K52A)* was made by PCR of CMK-1 (K52A) from *Pttx-1*::*CMK-1* (K52A) (Gift from Piali Sengupta) and cloning into pV6 with Nhe1 and Kpn1. pBM14 was injected at 25 ng/ul with Pmyo2::mCherry at 10 ng/ul to make *pzEx338*.

*Pglr-1*::*CMK-1*::*EGL-13 NLS* was made by PCR of *CMK-1*::*EGL-13 NLS* from *Pttx-1*::*CMK-1*::*EGL-13 NLS* (Gift from Piali Sengupta) and cloning into pV6 with Kpn1 and Sac1. pBM34 was injected at 25 ng/ul with Pttx-3::GFP at 50 ng/ul to make *pzEx370*.

### Fluorescence microscopy

#### GFP reporter quantitation

All GFP reporter imaging experiments were performed with a Carl Zeiss Axiovert M1 microscope with a 100x Plan Aprochromat objective (1.4 numerical aperture) with GFP and RFP filter cubes. Images were acquired with an Orca-ER charge-coupled device camera (Hamamatsu), using MetaMorph, version 7.1 software (Molecular Devices). All L4 animals were immobilized with 30 mg/ml 2,3-butanedione monoxamine (Sigma-Aldrich, St. Louis, MO) for 6–8 minutes before imaging. To quantitate GFP fluorescence, maximum intensity projections from Z-series stacks of 1 μm depth were taken from the PVC nucleus using MetaMorph software. Exposure settings were constant for each reporter. A region of interest was drawn around the nucleus and maximum pixel intensity was used for quantification. At least 20 animals were measured for each genotype and statistics were performed by Student’s *t* test (for two genotypes) or ANOVA with Tukey-Kramer *post hoc* correction (greater than two genotypes). Control genotypes were always assayed on the same day to normalize for daily fluctuations in fluorescence.

#### CMK-1::GFP subcellular localization

Fluorescence imaging of CMK-1::GFP was performed using a Zeiss LSM510 confocal microscope with a 63X objective (NA1.4). Images were acquired with a photomultiplier tube using Zeiss LSM 510 software. All L4 animals were immobilized with 30 mg/ml 2,3-butanedione monoxamine as described above. Z-series stacks were taken of PVC for each animal. Imaging settings were adjusted for each cell to optimize assessment of cytoplasmic vs. nuclear localization of CMK-1::GFP. Image acquisitions were taken blinded to genotype. Maximum projections of each image were used for scoring localization phenotypes. The nucleocytoplasmic distribution of CMK-1::GFP was categorized as either being enriched in the nucleus (CMK-1::GFP fluorescence in the nucleus > cytoplasm), equally distributed between the nucleus and cytoplasm (CMK-1::GFP fluorescence in the nucleus = cytoplasm) or enriched in the cytoplasm (CMK-1::GFP fluorescence in the nucleus < cytoplasm) by an experimenter blinded to the genotypes being scored. This scoring method was confirmed by another experimenter blinded to the genotypes. Statistics were performed using the Chi-squared test with *post hoc* corrections to assess significance vs. wild-type.

### Histamine chloride

L4 wild type and animals expressing *Pglr-1*::*HisCl1* were transferred onto plates with and without 10 mM histamine as previously described [39]. At zero, one, and four hours, animals were picked off plates and the GFP reporter was imaged as described above. Each time point was normalized to the zero hour and then to the corresponding time point of animals no exposed to histamine. Statistics were performed using Student’s *t* test at each time point comparing animals on histamine to animals off histamine.

### Behavior

Locomotion assays were performed as previously described [29, 60]. Briefly, wild type and animals expressing *Pglr-1*::*HisCl1* were placed on a plate with no food and allowed to acclimate for two minutes. Animals were then transferred to either a plate with or without 10 mM histamine (no food on either plate). Animals placed on histamine plates were exposed for 10 minutes. Animals were then observed for five minutes while reversals were counted manually. Behavioral assays were performed by an experimenter blind to the genotypes being observed.

### Real-time quantitative PCR (RT-qPCR)

Total RNA was isolated from ten 10 cm plates per genotype of mixed-stage animals by lysing in Trizol (Invitrogen) and extracting with an RNeasy Fibrous Tissue Mini kit (Qiagen) with on-column DNAse treatment. For each genotype, at least three independent RNA preparations were made alongside a corresponding wild type (N2) preparation to control for variation introduced by each preparation. cDNA from these RNA preps was synthesized using Superscript III Reverse Transcriptase (Invitrogen) and oligo d(T) primers. RT-qPCR was performed on the MX3000P real-time PCR machine (Tufts Center for Neuroscience Research) using the Brilliant SYBR Green QPCR Master Mix. The ΔΔCt method [61] was used to compute the relative amount of *glr-1* mRNA compared to two reference genes (*act-*1 and *ama-*1). Primers used for each gene (all oriented 5’ to 3’):

*glr-1*: F- CCGTTTAAACTTGCATTTGACC, R- ACAGACTGCGTTCACCATGT

*cmk-1* F- ATGCCCCTTTTTAAGCGACGG,

R- ACTGCATACATCTGACCGGCAT

*act-1* (DePina, 2011): F-CCAGGAATTGCTGATCGTATGCAGAA,

R-TGGAGAGGGAAGCGAGGATAGA

*ama-1* (Yan 2009): F- ACTCAGATGACACTCAACAC,

R- GAATACAGTCAACGACGGAG

SEM was calculated as previously described (Applied Biosystems). Statistical significance was determined using the Student’s *t* test on the geometric mean of the ΔCt values for each reference gene.

## Supporting Information

S1 Fig A GFP reporter under the control of *Pnmr-1* and the *unc-54* 3’UTR is not altered in *klp-4* and *glr-1* mutants. (A-B) Mean GFP fluorescence (Normalized) of reporter *Pnmr-1*::*NLS-GFP*::*LacZ*::*unc-54 3’UTR* in (A) wild type (n = 39) and *klp-4 (tm2114)* (n = 41) animals, and (B) wild type (n = 42) and *glr-1 (n2461)* (n = 34) animals is shown. Maximum GFP fluorescence was measured in the nucleus of the neuron PVC. Error bars represent SEM. Student’s *t* test was used to compare means. n.s. denotes no significant difference (p > 0.05).
(EPS)

S2 Fig The *unc-11*-induced decrease in the *glr-1* transcriptional reporter is not dependent on *glr-1*. Mean GFP fluorescence (Normalized) of reporter *Pglr-1*::*NLS-GFP*:*LacZ*::*unc-54 3’UTR* in wild type (n = 530), *unc-11 (e47)* (n = 149), *glr-1 (n2461)* (n = 397) and *unc-11 (e47); glr-1* (n2461) (n = 34) animals is shown. For all reporter imaging, maximum GFP fluorescence was measured in the nucleus of the neuron PVC. Error bars represent SEM. Values that differ significantly from wild type are indicated by asterisks above each bar. ANOVA with Tukey-Kramer *post-hoc* test was used to compare means. * p < 0.05, ** p < 0.01, *** p < 0.001. n.s. denotes no significant difference (p > 0.05).
(EPS)

S3 Fig The effects of histamine on the *glr-1* transcriptional reporter in wild type animals. Wild type animals were placed on plates with 10 mM histamine for one and four hours and mean GFP fluorescence of reporter *Pglr-1*::*NLS-GFP*::*LacZ*::*unc-54 3’UTR* was normalized to unexposed animals. n = 30 animals per condition. Error bars represent SEM. Student’s *t* test was used to compare means. * p < 0.05. n.s. denotes no significant difference (p > 0.05).
(EPS)

S4 Fig RT-qPCR analysis of *cmk-1* transgenes. Real-time qPCR of *pzIs29; cmk-1(oy21)* animals expressing *Pglr-1*::*CMK-1* (*pzEx333*) or *Pglr-1*::*CMK-1(K52A)* (*pzEx338*) comparing *cmk-1* expression in three biological replicates normalized to two references genes (*act-1* and *ama-1*). Student’s *t* test was used to compare mean ΔCt values.
(EPS)
